# Comparative Evaluation of Chair-Side Saliva Tests According to Current Dental Status in Adult Patient

**DOI:** 10.3390/dj9010010

**Published:** 2021-01-19

**Authors:** Chris Rahiotis, Panagiotis Mitropoulos, Afrodite Kakaboura

**Affiliations:** Department of Operative Dentistry, National Kapodistrian University of Athens, 11527 Athens, Greece; pgmitrop@gmail.com (P.M.); afrodite1@otenet.gr (A.K.)

**Keywords:** caries, saliva, DMFT, lactic acid, buffer capacity

## Abstract

Background: this cross-sectional study evaluated the correlation of commercial chair-side saliva tests with caries status in adults. Methods: teeth in 87 adults (20–40 years old) were clinically examined for carious lesions according to International Caries Detection and Assessment System (ICDAS) criteria. The Decayed-Missing-Filling-Tooth (DMFT) and Decayed-Missing-Filling-Surface (DMFS) indexes at D1 (lesions 1–6 according to ICDAS criteria) and D3 (lesions 4–6 according to ICDAS criteria threshold and the number of active lesions, according to the Lesion Activity Assessment (LAA)) criteria were measured. The saliva parameters measured by chair-side tests were stimulated and non-stimulated saliva flow rate, saliva consistency, saliva pH, saliva buffer capacity, and lactic acid production. The statistical analyses performed were Student t-test and Mann–Whitney U test at a = 0.05 significant level. Results: the low resting saliva pH was related to a high value of DMFT (D1) index (*p* = 0.007). Conclusions: among the saliva parameters measured, the values of low resting pH are associated with increased DMFT at threshold D1. None of the chair-side available saliva tests evaluated can accurately underline the tooth carious status.

## 1. Introduction

Tooth caries is a multifactorial disease due to an ecological imbalance between inorganic components of hard dental tissues and biofilms [1]. It is now believed that dental caries are considered to be a polymicrobial disease and do not follow the classical microbial Koch’s model [2]. It is further suggested that various species of microbes, such as mutans streptococci, lactobacilli, Actinomyces, bifidobacteria, and yeasts are associated with caries development [3].

The caries process also depends on the saliva parameters, the exposure to fluoride, the type of diet, and the type and frequency of oral hygiene habits [1]. The impact of an individual parameter in this dynamic relationship is not fully considered, with the development or progression of carious lesions not being predictable.

Τhe biological role of saliva is fundamental [4]. In particular, the ability of saliva to buffer acids is crucial for pH regulation in the oral cavity. Saliva includes different buffering systems that can regulate pH changes and neutralize the acids produced by cariogenic microorganisms [5]. These systems consist of bicarbonate, phosphate, and protein systems. Saliva also plays a fundamental role in maintaining the homeostasis of dental hard tissues due to the presence in its composition of calcium, phosphorous, and other inorganic ions in its composition [4]. Furthermore, hyposalivation may be accompanied by shifts in specific microbial species, toward a highly acidogenic microflora [4]. A commercial chair-side quick and easy saliva-testing examination tool has been on the markets (Saliva Check Buffer, GC, Tokyo, Japan). It measures the consistency, pH, flow, and buffering capacity of the saliva. 

The Clinpro Cario-L-Pop (3M ESPE, AG, Seefeld, Germany) is a semi-quantitative method for calculating lactic acid production from the microbial plate of the dorsal surface of the tongue. This technique is essentially based on the ecological theory of plaque, namely the prevalence of acid-producing bacteria leads to the reversal of the balance in the demineralization-remineralization cycle, resulting in the development or progression of carious lesions [6]. Lactic acid production is related to the demineralization of dental hard tissues [7]. Based on lactic acid production and the resulting change in the color index, the patient is classified at different caries risk group. It was previously evaluated and can identify changes in the oral microbiome and, thus, could be used to monitor the impact of interventions [8,9,10,11].

The ability to predict the formation or progression of carious lesions, based on the individual (or all of the) parameters involved in the disease would be a particularly useful diagnostic tool for adopting and identifying specific preventative measures, as well as distinguishing between the limits of preventive and invasive dentistry. In this direction, chair-side tests that have been developed and are commercially available attempt to quantify the parameters associated with caries. An advantage of using these tests for evaluating saliva to identify the caries risk is that these record results immediately after the collection of saliva. Moreover, these tests could be used in the everyday clinical practice, without the support of complex and expensive laboratory procedures [12]. 

However, it is unclear if the records of these chair-side tests are reliable and clinically correlated with the true clinical caries status at a patient level. To date, no information has been devoted to the reflection of the clinical assessment of the number of the lesions, as well as, at the same time, the activity of a lesion with the records of these tests. Thus, the present study aimed to evaluate, comparatively, the recordings of commercially available chair-side saliva tests with current caries status in adults. The null hypothesis was that the formulations tested did not show differences in recordings of saliva tests according to the carious status in the examined oral environments.

## 2. Materials and Methods 

Eighty-seven (*n* = 87) patients participated in this clinical study. The patients were treated at undergraduate and postgraduate clinics of the Dental School of Athens, National and Kapodistrian University of Athens. The study was approved by the Committee of Ethics of the Dental School of the University (protocol number #47), and all patients who participated in the study were informed and gave their prior written consent. The inclusion criteria for patient participation in the study were: age of patients between 20 and 40 years; the presence of at least 20 natural teeth in the oral cavity; the absence from the medical history of diseases affecting saliva; not taking medications that affect the flow of saliva; not taking antibiotics and antimicrobial drugs for 15 days before the clinical examination; no active orthodontic therapy, no professional application of fluoride during the past 6 months, and the non-use of oral hygiene products with antimicrobials (e.g., chlorhexidine).

The examination was performed at the same time for all patients at midday (10 a.m. to 11 a.m. in the morning). The examination was done by the same examiner (P.M.) who is trained and calibrated. The examiner was trained with educational software accompanying the International Caries Detection and Assessment System (ICDAS) scoring system (ICDAS training software) to use these criteria before the clinical examination of the teeth. Further training was accomplished with a second examiner (C.R.) who also trained and validated with the ICDAS criteria. The two examiners scored pilot ten teeth independently, and then discussed the scores, presenting a degree of agreement. The scoring of ten other teeth was repeated after one week, separately by the two examiners, and the two examiners came in full agreement. Finally, at a different time, the two examiners scored independently 20 other teeth, and the agreements were substantial (kappa = 0.9). An oral examination was conducted, and the condition of teeth, gums, and fillings were registered. Thereafter, the teeth were professionally polished, and the presence of carious lesions was recorded, based on the visual assessments. Carious lesions were characterized as active or inactive, and classified according to the ICDAS criteria, in any surface, into 7 grades (0–6). Additionally, for each patient, the number of potential activities of the lesions, based on the Lesion Activity Assessment (LAA), was determined and recorded [13]. Based on this system, each lesion was characterized by its extent, location, and texture, by taking one digit according to the features it displayed. Then, the digits of the individual classes were summed, and if the sum was greater than 7, the lesion was characterized as possibly active.

Immediately after, the caries activity was measured with the Clinpro Cario-L-Pop test (3M ESPE, AG, Seefeld, Germany). This formulation is a semi-quantitative method of determining the metabolic activity of carious microorganisms. The microbial plate sample from the dorsal surface of the tongue, by rotating the specific swab pre-impregnated into glucose, was collected. The swab was then immersed in a lactate dehydrogenase solution to catalyze the conversion of lactic acid to pyruvic acid. After 2 min, the color of the stylus was compared to the nine-step color guide included in the formulation. According to the manufacturer’s instructions, grades 1–6 correspond to normal caries activity, and high for grades 7–9, respectively.

The next clinical step was the assessment of qualitative and quantitative saliva characteristics. At this stage, the following characteristics were evaluated and recorded: (a) saliva composition; (b) saliva pH; (c) saliva flow in a resting state; (d) saliva flow in a stimulated state; and (e) saliva buffer capacity. Initially, the patient was asked to bring the tongue to the palate to reach the saliva lying in the mouth of the oral cavity. The viscosity of saliva was evaluated in the following grades: sticky and non-sticky. Subsequently, the patient’s lower lip was dried using gauze and observed under strong illumination to record the creation of saliva droplets in the mouths of the minor salivary lip glands [14]. The presence of saliva droplets in less than one minute was recorded as a high saliva flow in a resting state, with a longer time corresponding to a low saliva flow.

Then, to calculate the saliva pH in a resting state, the patient was asked to pour the saliva into a plastic cube in order to wet the pH-specific paper indicator contained in the GC Saliva-Check Buffer (GC, Tokyo, Japan) formulation. The measurement indicator was compared to the corresponding color guide to record the pH value. PH values above 6.8 correspond to normal saliva, while values below 6.6 were characterized as acidic.

Next, to calculate the saliva flow in a stimulation state, the patient was asked to chew the paraffin wax. After 30 s, the patient exfoliate the saliva produced in sputum in order to remove the saliva accumulated in the salivary gland pores before the onset of the chewing stimulus. The chewing of the paraffin cube continued for 5 min, during which the produced saliva was collected in a plastic volumetric tube. Total production of less than 5 mL was characterized as low saliva production in a state of excitation. A total saliva volume of more than 5 mL was high. After that, the buffering capacity of the collected saliva was recorded by the specific marker included in the GC Saliva-Check Buffer (GC, Tokyo, Japan) package. The absorbent paper received red, blue, and green tint, corresponding to 0, 2, and 4 degrees. Total sum, 0–9, corresponded to low buffering, and 10–12 corresponded to normal capacity, respectively. 

**Statistical Analysis**

The The Decayed-Missing-Filling-Tooth (DMFT) and Decayed-Missing-Filling-Surface (DMFS) index was calculated in each patient at D1 (included lesions 1–6, according to ICDAS criteria) and D3 threshold (lesions 4–6, according to ICDAS criteria). At the D1 diagnostic threshold, the 0 = absent of carious lesions, 1 = lesions grater to 1 based on ICDAS criteria, and at the D3 diagnostic threshold, 0 = 1–3 ICDAS grades were recorded as no caries, and 4–6 and were recorded as caries, respectively.

The results of the impact of each parameter on DMFT (D1) and DMFT (D3) clinical indexes were subjected to Student t-test statistical analysis at a statistical significance level of a = 0.05. The results of the impact of each parameter on the clinical indexes DMFS (D1) and DMFS (D3), as well as the number of potentially active lesions, were subjected to a Mann–Whitney U test statistical analysis, with a statistical significance a = 0.05. Logistic regression analyses were run to determine the association of LAA values with saliva parameters. The odds ratio was calculated with a dependent variable of the high number of potentially active lesions, using as a limit the median of the distribution of the number of active lesions. The level of statistical significance was set at a = 0.05. The statistical analysis was done using SPSS (Version 20.0.0, IBM, Chicago, IL, USA). 

## 3. Results

The results of the impact of the individual saliva test parameters on DMFT (D1) and DMFT (D3) are presented in Table 1. 

Statistical analysis showed that abnormal saliva pH recording in a resting state was associated with an increase in DMFT (D1) relative to normal saliva pH (*p* = 0.007). 

The impact of the individual saliva test parameters on DMFS (D1) and DMFS (D3), as well as the number of potentially active lesions, are given in Table 2. 

There was no correlation among the saliva parameters and DMFS and active lesions values.

The results of the multivariate analysis for the high number of potentially active lesions are presented in Table 3. 

There was no risk factor for the existence of a high number of potentially active carious lesions at a statistically significant level.

## 4. Discussion

The null hypothesis of this study was rejected. The commercially available saliva tests did not accurately indicate the real tooth carious status. Only the parameter low resting saliva pH was related to a high value in DMFT at the D1 threshold and the high number of possible active carious lesions, respectively. 

The most used clinical index in epidemiological studies is the DMF at the D3 diagnostic threshold; it is easily applicable and records the lesions at a distinct stage of cavity threshold. A major drawback is that the use of this index, at the D3 diagnostic level, does not consider the recording of carious lesions in stages before the cavity formation. This disadvantage is considered important nowadays because a minimally invasive philosophy predominates, and for this reason, there is a need to recognize the lesions at initial stages. For these reasons, in the present clinical study, the DMFT and DMFS indexes were recorded at the D1 diagnostic threshold [15]. 

Even in this case, the use of the DMF has some drawbacks. It records the total history of the dental arch, and there is the result of summing the recordings from carious lesions, restorations, and missing teeth or surfaces respectively [16]. Based on the aforementioned, although this clinical index is widely applicable, its use for the evaluation of the caries risk and the related saliva parameters remains problematic. For this reason, in the present study, all potentially active carious lesions based on the ICDAS and the related LAA activity determination system were recorded [17].

In our study, the saliva characteristics were studied based on the Saliva-Check Buffer Kit (GC, Tokyo, Japan). Indeed, saliva pH and flow, in rest and stimulation, is an individualized feature, and is characterized by biological variability. It seems that it is not reliable to clearly define the minimum normal salivary flow rate, as carious lesions can also develop in people with normal saliva flow [17]. It was reported that the decreased saliva flow in a resting state is more important than the saliva flow in a state of stimulation [18]. In this study, the saliva flow, in rest and stimulation, does not appear to have an impact on any of the caries parameters considered. These findings come in agreement with previous studies on the stimulated salivary flow in different age groups of patients [19,20,21,22]. It was also previously reported that there is no correlation of clinical DMFT and DMFS indicators at the D3 threshold, with the saliva measured in rest and stimulation conditions [23]. On the contrary, in that study, a correlation between the caries activity and the saliva flow was indicated. The methodology used in our study to measure the rest saliva flow has a practical difficulty in determining the distinction between droplet formations or not, which is an arbitrary unit of measurement. This test is based on the view of saliva production by the minor salivary glands of the lower lip region as a clear measure of saliva production at rest. The only available report on the use of this technique relates to a study of the impact on the demineralization of enamel in children. In that study, the low production of saliva in a state of relaxation, based on the instructions of the Saliva-Check Buffer Kit (GC, Tokyo, Japan) formulation, was associated with extensive loss of dental substance on pre-existent demineralized enamel surfaces [24].

In regards to the viscosity of saliva, the results indicate that the high value of the DMFS (D3 threshold) was not correlated with sticky saliva (p = 0.076). The viscosity of saliva and the relationship with the clinical dental caries has not been examined, previously. The only report in the literature referred to the presence of reduced oral mucosal motility in patients with severe medical history and high caries risk [18].

The present study revealed that the abnormal pH of resting saliva leads to a significant increase in the DMFT (D1) score. This finding is consistent with previously reported data in which a negative correlation of the saliva pH value existed with the total number of initial lesions (grades 1 and 2, according to ICDAS) [23]. Moreover, it has been reported that there was a correlation between the severity of lesions and abnormal pH on demineralized enamel surfaces in children [24]. However, other studies showed conflicting results, either negative or a positive correlation of the stimulated saliva pH and carious lesions at the D3 threshold [25,26]. 

The measurement of the buffer capacity of saliva is the greatest diagnostic challenge due to the complex saliva pH regulating systems. In previous work, no statistically significant difference in the buffer capacity between individuals who experienced new carious lesions in the 4-year follow-up and those without new lesions was reported [26]. A study investigated the correlation of the buffer capacity measured with the Saliva Check Buffer with the DMFT and DMFS indexes at the D3 threshold, as well as with the caries activity [23]. Those results indicated a correlation of low buffer capacity with all potentially active lesions (grades 3 and 4, according to ICDAS criteria), but no correlation with the DMFT (D3) and DMFS (D3) clinical scores. Those results are in line with the findings of our work. Additionally, in the present study, the lack of statistically significant differences is confirmed in both the D1 and D3 diagnostic thresholds.

The Clinpro Cario-L-Pop test (3M ESPE, AG, Seefeld, Germany) is suggested to be able to detect changes in the microbial flora of the mouth [8,9]. A practical, clinical problem, addressed in the present study, is the uneven coloring of the surface to be assessed, as the end portion of the stylus was colored more clearly. This clinical problem was reported in a study in which the repeatability of the method between different examiners were low [8]. In another study, in which a large number (*n* = 771) of pediatric dental patients was examined, it was reported that the average and high values of Clinpro Cario-L-Pop tests (3M ESPE, AG, Seefeld, Germany) were not related to the number and the severity of carious lesions, and that method also displayed a significant number of false positives [27]. However, according to another study, the combination of the DMFT and the Clinpro Cario-L-Pop test may help identify orthodontic patients with high caries risk [28]. A point of concern in the study was that it was not described at which the diagnostic threshold was used for the calculation of DMFT and DMFS values. 

The mapping of the parameters examined in this study and the clinical picture is instantaneous, since the above-mentioned parameters are not necessarily constant over time. Consequently, the results of both the present and the rest of the relevant studies should be considered, with the awareness of the variability of the parameters associated with the carious disease. Moreover, as manufacturers attempt to attribute the ability to determine the caries risk, it is necessary to monitor the patients after a reasonable period. The calculation of the predictive value of each diagnostic method determines its contribution to the prognosis of the caries progression. The design of the study should have the characteristics of the prospective studies, including the recording of the initial state and the recall, after a given interval time.

## 5. Conclusions

The results of the present study show that none of the saliva parameters examined were associated with a change in all individual clinical indexes. Moving forward, the protocol in the patient recall phase will result in the predictive value of the available diagnostic tools, to ultimately point out their usefulness in the determination of the risk of caries disease.

## Figures and Tables

**Table 1:** Distribution of sample individuals per parameter according to DMFT (D1) and DMFT (D3).

		DMFT(D1)		DMFT(D3)	
	N	Mean Value (±SD)	*p*	Mean Value (±SD)	*p*
**Resting Saliva flow**					
Low	21	16.24 (7.17)	0.47	14.57 (7.5)	0.81
High	66	17.18 (6.57)		14.86 (7.36)	
**Stimulated Saliva flow**					
Low	23	15.21 (5.5)	0.49	13.08 (6.3)	0.48
High	64	17.57 (7.0)		15.4 (7.65)	
**Saliva consistency**					
Sticky	4	23 (3.92)	0.25	22.5 (3.7)	0.16
No sticky	83	16.67 (6.67)		14.42 (7.29)	
**Saliva pH at rest**					
Low	37	17.89 (7.84)	0.007	15.68 (8.41)	0.33
Normal	50	16.26 (5.67)		14.14 (6.47)	
**Buffer Capacity**					
Low	43	17.02 (6.85)	0.55	14.56 (7.77)	0.32
Normal	44	16.89 (6.61)		15,02 (7.00)	
**Lactic Acid**					
High	56	16.68 (6.59)	0.39	14.88 (6.92)	0.075
Low	31	17.45 (6,95)		14.65 (8.19)	

**Table 2:** Distribution of sample individuals per test parameter according to the DMFS index (D1), the DMFS index (D3) and the number of potentially active lesions.

		DMFS(D1)		DMFS(D3)		Active Lesions	
	N	Mean Value (±SD)	*p*	Mean Value (±SD)	*p*	Mean Value (±SD)	*p*
**Resting Saliva flow**							
Low	21	50.67 (30.85)	0.804	47.52 (31.09)	0.758	4.43 (5.31)	0.212
High	66	48.90 (28.67)		45.23 (29.47)		6.18 (8.86)	
**Stimulated Saliva flow**							
Low	23	40.35 (22.86)	0.119	37.17 (23.83)	0.133	5.04 (4.15)	0.523
High	64	52.55 (30.47)		48.88 (31.13)		6.02 (9.19)	
**Saliva consistency**							
Sticky	4	76.25 (33.06)	0.109	75.25 (33.01)	0.076	4.00 (2.16)	0.977
No sticky	83	48.02 (28.41)		44.36 (28.3)		5.84 (8.32)	
**Saliva pH at rest**							
Low	37	54.22 (34.31)	0.386	50.65 (34.37)	0.370	7.41 (10.55)	0.142
Normal	50	45.7 (24.16)		42.18 (25.38)		4.54 (5.58)	
**Buffer Capacity**							
Low	43	48.72 (31.53)	0.575	44.77 (32.52	0.575	6.72 (10.28)	0.608
Normal	44	49.09 (26.73)		46.77 (27.00)		4.82 (5.28)	
**Lactic Acid**							
High	56	48.29 (27.19)	0.856	45.02 (27.67)	0.965	6.23 (9.34)	0.993
Low	31	51.19 (32.50)		47.16 (33.49)		4.90 (5.40)	

**Table 3:** Odds ratios (OR) and 95% confidence interval (CI) derived from logistic regression.

Dependent Value	Independent	OR	95% CI	*p*
High number of active lesions	Constant	0.849		0.79
	Low buffer capacity	1.119	0.438–2.857	0.813
	Low Saliva pH at rest	1.24	0.478–3.215	0.658
